# Genome-Wide siRNA-Based Functional Genomics of Pigmentation Identifies Novel Genes and Pathways That Impact Melanogenesis in Human Cells

**DOI:** 10.1371/journal.pgen.1000298

**Published:** 2008-12-05

**Authors:** Anand K. Ganesan, Hsiang Ho, Brian Bodemann, Sean Petersen, Jayavani Aruri, Shiney Koshy, Zachary Richardson, Lu Q. Le, Tatiana Krasieva, Michael G. Roth, Pat Farmer, Michael A. White

**Affiliations:** 1 Department of Dermatology, University of California Irvine, Irvine, California, United States of America; 2 Department of Biological Chemistry, University of California Irvine, Irvine, California, United States of America; 3 Department of Cell Biology, University of Texas Southwestern Medical Center, Dallas, Texas, United States of America; 4 Department of Biochemistry, University of Texas Southwestern Medical Center, Dallas, Texas, United States of America; 5 Department of Dermatology, University of Texas Southwestern Medical Center, Dallas, Texas, United States of America; 6 Beckman Laser Institute, University of California Irvine, Irvine, California, United States of America; 7 Department of Chemistry, University of California Irvine, Irvine, California, United States of America; Stanford University School of Medicine, United States of America

## Abstract

Melanin protects the skin and eyes from the harmful effects of UV irradiation, protects neural cells from toxic insults, and is required for sound conduction in the inner ear. Aberrant regulation of melanogenesis underlies skin disorders (melasma and vitiligo), neurologic disorders (Parkinson's disease), auditory disorders (Waardenburg's syndrome), and opthalmologic disorders (age related macular degeneration). Much of the core synthetic machinery driving melanin production has been identified; however, the spectrum of gene products participating in melanogenesis in different physiological niches is poorly understood. Functional genomics based on RNA-mediated interference (RNAi) provides the opportunity to derive unbiased comprehensive collections of pharmaceutically tractable single gene targets supporting melanin production. In this study, we have combined a high-throughput, cell-based, one-well/one-gene screening platform with a genome-wide arrayed synthetic library of chemically synthesized, small interfering RNAs to identify novel biological pathways that govern melanin biogenesis in human melanocytes. Ninety-two novel genes that support pigment production were identified with a low false discovery rate. Secondary validation and preliminary mechanistic studies identified a large panel of targets that converge on tyrosinase expression and stability. Small molecule inhibition of a family of gene products in this class was sufficient to impair chronic tyrosinase expression in pigmented melanoma cells and UV-induced tyrosinase expression in primary melanocytes. Isolation of molecular machinery known to support autophagosome biosynthesis from this screen, together with *in vitro* and *in vivo* validation, exposed a close functional relationship between melanogenesis and autophagy. In summary, these studies illustrate the power of RNAi-based functional genomics to identify novel genes, pathways, and pharmacologic agents that impact a biological phenotype and operate outside of preconceived mechanistic relationships.

## Author Summary

Aberrant pigment regulation correlates with skin disorders, opthalmologic disorders, and neurologic disorders. While extensive studies have identified regulators of mouse coat color, the regulation of human skin phenotypic variation is less well understood. To give a broader picture of the molecular regulators of melanogenesis in human cells, we used a genome-wide siRNA functional genomics approach to identify 92 novel regulators of melanin production in heavily pigmented MNT-1 melanoma cells. Our screen identified several genes that converge to regulate tyrosinase, the rate-limiting step in pigment production, in both MNT-1 cells and primary melanocytes. Some of the identified genes were selectively active in different genetic backgrounds, suggesting that they may regulate human phenotypic variation. Small molecule inhibition of a family of novel pigment regulators was sufficient to impair pigment production in melanocytes. Additionally, our screen identified molecular machinery known to support autophagosome biosynthesis as putative regulators of melanogenesis. *In vitro* co-localization studies and autophagy-deficient mice provided evidence that normal melanogenesis requires the same molecular machinery used by the autophagy pathway. Taken together, these results illustrate the utility of genome wide siRNA screening approaches for identifying genes, novel pharmacologic agents, and pathways that regulate differentiated cellular phenotypes.

## Introduction

Significant effort has been focused on identifying the molecular etiology for pigment variation in skin [1]. 127 mouse coat color genes have been identified [2], 68 of these genes have human homologues, and 29 of these homologues impact pigmentation in humans. Genetic mapping studies have identified a limited set of genes responsible for skin and eye color variability [3]. Pigment production involves the concerted actions of transcriptional, translational, and intracellular trafficking machinery [4]. MITF, the master regulator of melanogenesis in the mouse hair follicle [5], activates the transcription of tyrosinase, the rate limiting step in melanogenesis [5]. Tyrosinase is translated in the endoplasmic reticulum and is glycosylated in the Golgi apparatus [6]. Tyrosinase activity is restricted to the melanosome, a melanin specific organelle of poorly defined origin [7],[8]. While the subtle variation in human skin color is thought to be the result of the complex interaction of multiple genes, the majority of mouse mutants described have segmental or complete absence of pigment [9]. Recent studies have identified partial loss of function mutations that impact the shade of melanin in zebrafish and human skin [10], but the spectrum of gene targets that regulate pigment shade is unknown. Melanin is expressed in different end organs conferring different functions. Melanin protects the skin, eyes [1], and brain from toxic insults [11]. Melanin in the inner ear impacts sound conduction [12]. Loss of melanin is thought to play a role in the etiology of age related macular degeneration [13] and Parkinson's disease [14]. Additionally, melanin is aberrantly regulated in human skin disorders such as vitiligo and melasma. Harnessing the molecular mechanisms that regulate melanogenesis to selectively modulate melanin production in the skin, eye, or brain could lead to novel treatments for multiple human pathologies. Pharmacologic modulation of melanin production has primarily focused on identifying inhibitors of tyrosinase, the rate limiting step in pigment production [15]. Currently utilized tyrosinase inhibitors are clinically effective, but are carcinogenic in animal studies [16]. Pharmacologic agonists that stimulate pigmentation in human tissues remain to be identified. A better understanding of the molecular network governing pigment production in the human epidermis is indicated to aid design of agents that inhibit or stimulate pigmentation in human skin.

## Results/Discussion

Studies to determine the key molecular regulators of melanogenesis in human melanocytes have been hampered by the innate fragility of these cells and the fact that they produce scant amounts of pigment in culture [17],[18]. To identify novel regulators of melanogenesis in human cells, we utilized MNT-1 melanoma cells to screen a genome-wide synthetic siRNA library for single-gene loci that support melanocyte pigmentation. MNT-1 cells produce substantial amounts of melanin in culture, have a gene expression profile that is most similar to normal melanocytes [19], and have been used by others to identify pigment regulatory mechanisms that govern normal melanogenesis [20]–[23]. We employed a previously described [24] Dharmacon siRNA library of 84,508 siRNAs corresponding to four unique siRNA duplexes, targeting each of the 21,127 unique human genes arrayed in a one-gene/one-well format on 96 well microtiter plates. A spectrophotometric melanin quantitation assay was coupled with an ATP-dependent luminescence cell viability assay (CellTiter-Glo) to eliminate siRNAs that decrease melanin production as a consequence of impacts on either cell proliferation or cell survival. Using tyrosinase depletion as a positive control, we determined that a 5-day post-transfection incubation period was optimal for quantitative detection of impaired melanin production (Figure S1). Other studies demonstrated that the cell titer glo assay did not interfere with the spectrophotometric quantitation of melanin (data not shown). In order to identify genes that impact both pheomelanin and eumelanin production, we measured melanin content at 405 nm [25], a wavelength at which both pheomelanin and eumelanin absorb light.

Raw A_405nm_ absorbance values were normalized to internal reference samples on each plate to permit plate-to-plate comparisons. This analysis was followed by normalization to the experimental mean for each well location calculated from the full data set in order to control for variations in pigment due to plate position effects. Similarly adjusted luminescence values from the multiplexed viability assay were used to generate “normalized absorbance ratios” for each well (Table S1). The distribution of the means of these values from duplicate analyses is shown in Figure S1B. Previous studies have identified 68 genes that regulate pigment production in human cells. Initial examination of our dataset determined that siRNAs directed towards 13 of these 68 genes impaired melanin accumulation without impacting melanocyte survival or proliferation when depleted in these assays, with tyrosinase itself scoring with one of the lowest ratios (2.5 standard deviations below the mean; Table S2). Our current siRNA screening protocol relies on siRNA design algorithms to identify effective siRNA sequences and utilizes a single endpoint assay to identify siRNAs that impact pigment production. Genes whose function is not inhibited by selected siRNAs either secondary to the long half-life of the corresponding protein or secondary to poor siRNA sequence selection would not be identified in our screening approach. Identification of several known regulators of melanogenesis by our screening protocol does give confidence that our approach is sufficiently robust to identify novel regulators of pigment production.

To facilitate the identification of novel genes that significantly impact melanogenesis, a cutoff of 2 standard deviations below the mean was used to select a candidate hit list. 98 genes were identified as regulators of melanogenesis by our screening approach. Of these 98 genes identified in the primary screen, only 6/98 genes (marked in red, Table 1) exhibited aberrant expression in MNT-1 cells as compared to normal melanocytes [19], indicating that the screen identified a large number of genes that likely impacted melanogenesis in both primary melanocytes and MNT-1 cells. Two of the genes identified in our screening approach were more recently eliminated from the Refseq database, and were not subject to detailed further evaluation.

**Table 1:** Candidate Pigmentation Genes.

Category	Symbol	Comments	Motifs
**autophagy**	MAP1LC3C		MAP1_LC3
	WIPI1	expressed in melanoma cell autophagosomes	WD40
	GPSM1		GoLoco
**GPCR**	GNG2		GGL
	GPR113		GPS, 7tm_2
	EDNRA	linked to migraine resistance	7tm_1
	OR4F15		7tm_1
	EDG7		7tm_1
	GPR92		7tm_1
	AGTR2	linked to mental retardation	7tm_1
	GRM7		ANF_receptor, NCD3G, 7tm_3
	GPR84		7tm_1
	P2RY1		7tm_1
**transcription**	PLAGL1	mutation causes Beckwith-Wiedeman syndrome	Znf_C2H2
	EZH1		SANT, SET
	TEF	maps to pigment mutations in mice	BRLZ
	GATAD2A		
	ILF2		DZF
	SMARCC2		CHROMO, SWIRM, SANT
**pigment**	**TYR**	**Albinism**	**Tyrosinase**
	BMP1		ZnMc, CUB, EGF_CA
**phospholipid**	PNPLA4		Patatin
**signaling**	ZFYVE1		FYVE
	ITPK1	maps near SNPs linked to pigmentation	Ins134_P3_kin
	PLCXD1		PLCc
	NRGN		IQ
	PLEKHA1	linked to age related macular degeneration	PH
**Ras family**	RAB4A		RAB
**GTPase**	**HRASLS**		**NC**
	ARL4A		ARF, small_GTPase
	ZDHHC9	linked to mental retardation	zf-DHHC
	C5ORF5		RhoGAP
	ARHGEF11		PDZ, RGS, PH, RhoGEF
	KLC4		Rab5-bind, TPR
**protease**	**SERPINB2**		**SERPIN**
**inhibitor**	WFDC8		WAP, KU
	**SERPINE1**		**SERPIN**
	SERPINB1		SERPIN
**metabolism**	NT5E		Metallophos, 5_nucleotid_C
	G6PC3		AcidPPc
	UROD	mutation causes porphyria cutanea tarda	URO-D
	HPD	mutation causes tyrosinemia type III	glyoxalase
	ALDH9A1		Aldedh
	PLTP		BPI1, BPI2
	MSRA	downregulated in vitiligo (hypopigmentation)	PMSR
	SMOX		Amino_oxidase, DAO
	UEVLD		UBCc, Ldh_1_N, Ldh_1_C
	GMPPB		NTP_transferase, Hexapep
	**ALDH1A1**	**expression lost in Parkinson's disease**	**Aldedh**
	MGC4172		adh_short, Epimerase, KR
	ENO2		Enolase_N, Enolase_C
**protein**	NLK		S_TKc
**phosphorylation**	PKN2		Hr1, C2, S_TKc, S_TK_X
	RIOK1		RIO
	PPP1R15A	expression lost in melanoma transformation	
	PPP2CB		PP2Ac
**helicase**	RTEL1		DEXDc, HELICc
	LOC389901		Ku, SAP DNA bd
**peptidase**	ARTS-1		Peptidase_M1
	KLK13		Tyrp_SPc
	LYZ	Amyloidosis	LYZ1
	ADAM19		Pep_M12B_propep, Reprolysin, DISIN, ACR, EGF_2
	CPZ		FRI, Zn_pept
	TRY1		Tryp_SPc
	SENP1		DSS1_SEM1
	SHFM1	split hand/foot malformation	Peptdiase_C48
**translation**	EEF1A1		GTP_EFTU, GTP_EFTU_D2, GTP_EFTU_D3
	VARS2		tRNA-synt_1, Anticodon_1
**other**	NPM3		nucleoplasmin
	STX18		syntaxin
	KRTAP4-11		Keratin_B2
	FGF23	overexpressed in hyperpigmentation syndrome	FGF
	SFRS2		RRM
	SLC17A5	mutation causes Salla disease	MFS_1
**unknown**	USHBP1		
	UBE2V1		UBCc
	TEX11		TPR_2
	TANC2		ANK, TPR
	FATE1		
	LRRC1		LRR
	RTN3		Reticulon
	SPATA22		
	ETAA1	tumor antigen, melanoma of soft parts	
	c12orf49		
	FAM125B		
	HSPC049		WD40
	AFAP1L2		PH
	FLJ41423		
	**MAGEA6**	**melanoma antigen**	**MAGE**
	MUC3b		EGF, SEA
	C1orf194		NuA4
	FAM89B		

Our siRNA-based screening approach identified 98 siRNAs that significantly inhibited pigment production. 4 of these 98 genes did not retest, while two of the 98 genes were removed from the Refseq database. Gene Ontology databases were used to segregate the 92 remaining genes into function classes and identify conserved domains within the corresponding proteins. Gene ontology databases, OMIM, and Pubmed searching was utilized to identify associations between our genes and human diseases. Genes in our candidate list that are aberrantly expressed in MNT-1 cells as compared to normal melanocytes are shown in bold.

**Figure 1:**
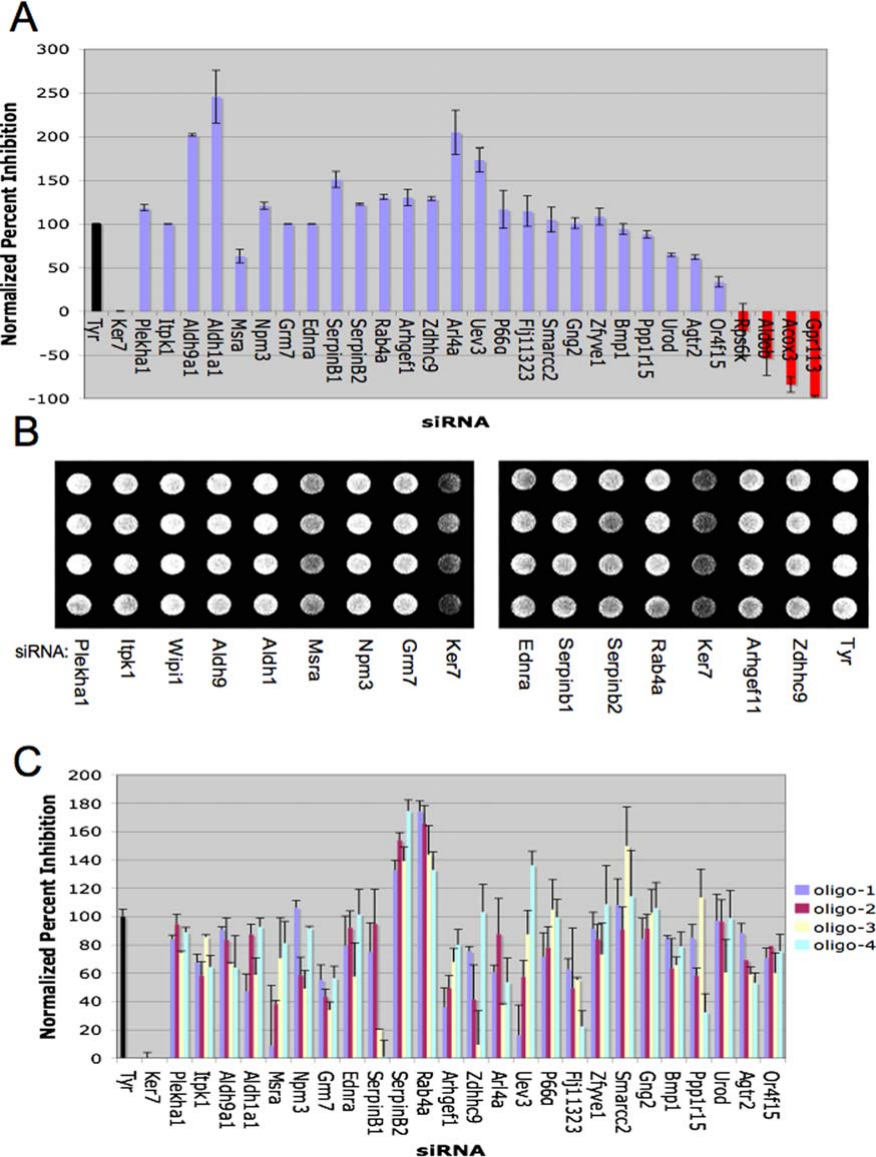
Validation of novel gene products supporting melanogensis. A) MNT-1 cells were transfected with the indicated siRNA pools (50 nM final concentration) targeting 35 of the 94 positive regulators of melanogenesis identified in the primary screen. siRNAs targeting Ker7, a gene that does not impact pigment production, were used as a negative control (black bar). A normalized percent inhibition calculation [26] was employed to compare the consequences of each siRNA pool on pigmentation with that observed upon depletion of tyrosinase. Bars represent mean and s.e.m. for n = 3. Red bars indicate failure to significantly suppress pigmentation. The results of the analysis of these 35 genes are shown in this panel (those genes that were not putative autophagy regulators) and in Figure 3 (putative autophagy regulators). A summary of the results for all 35 genes is shown in Table S4. B) A light micrograph of a representative opaque-walled, clear-bottomed 96-well microtiter plate containing MNT-1 cell monolayers 7 days post transfection with the indicated siRNAs is shown. C) Four independent siRNAs targeting the indicated genes (see Table S3 for siRNA sequence information) were separately tested for the capacity to suppress pigmentation as in (A). Associated p-values (student's t-test) are reported in Table S4.

Individually synthesized, pooled siRNAs directed against 35 of the 96 remaining genes selected from the primary screen, as described above, were retested to determine the false-positive rate (Table S4). These genes were randomly selected from the putative target list. To more precisely control for the efficacy of siRNA transfection and to correct for the background absorbance of MNT-1 cells, the ability of each target siRNA to inhibit pigment production was compared to the ability of tyrosinase siRNA to inhibit pigment production using a normalized percent inhibition calculation [26], and relative pigmentation was assessed visually prior to cell lysis (Figure 1A,B). A Keratin 7 siRNA pool that did not impact pigment production was utilized as a negative control. Four siRNA pools failed to significantly impact pigment production upon retesting and were eliminated from further analysis (Figure 1A, Table S4), giving an estimated false discovery rate of 12.1%. To validate that our candidate siRNAs inhibit the expression of the gene of interest, we utilized quantitative RT PCR to examine if a random selection of candidate siRNA pools inhibited the expression of the appropriate target gene (Figure S2). These results validate that the siRNAs selectively impact the expression of the cognate target gene, although this may not conceivably hold true for all of the siRNAs used in our screen. To eliminate siRNA pools with off-target effects on melanogenesis [24], the four siRNAs comprising each siRNA pool were retested individually. We found that at least two independent siRNAs against each target gene significantly inhibited pigment production (Figure 1C, Table S4), suggesting that pigmentation phenotypes are not a common consequence of siRNA off-target phenomena. Together, these studies demonstrate that the genome wide siRNA screening platform accurately identified gene targets that specifically impact pigment production.

**Figure 2:**
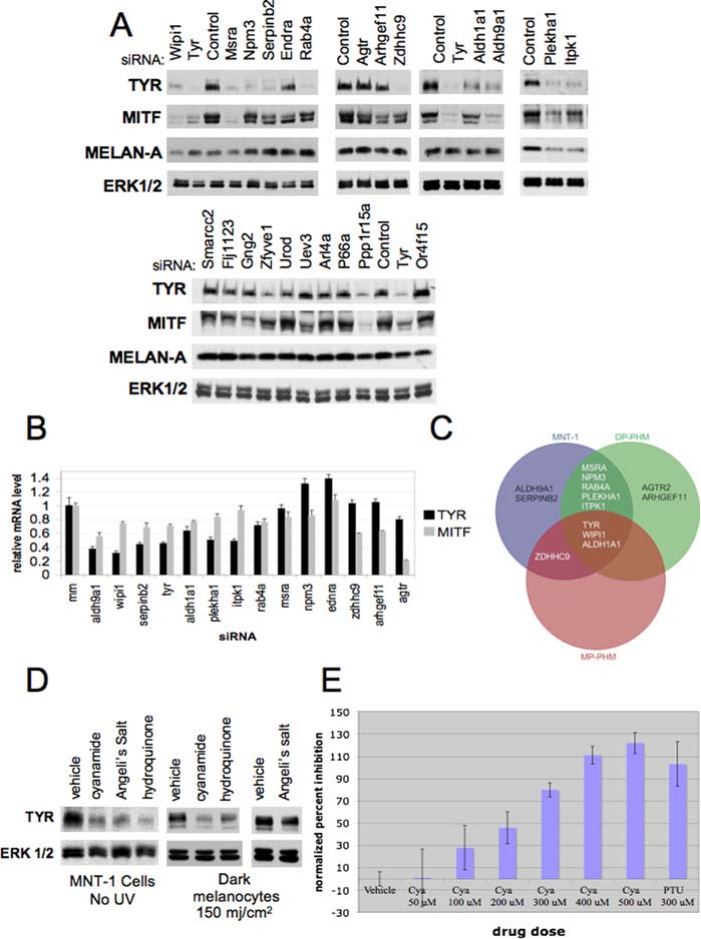
Novel, pharmaceutically-tractable melanogenesis gene networks converge on tyrosinase expression. A) 4 days post transfection with the indicated siRNAs, MNT-1 whole cell lysates were prepared and analyzed by immunoblot for the indicated proteins. A non-targeting siRNA was used as a transfection control (Control). ERK1/2 is shown as a loading control. B) Those siRNAs that inhibited tyrosinase accumulation were examined for consequences on tyrosinase and MITF gene expression by quantitative RT-PCR. 72 hours post transfection with the indicated siRNAs, equal numbers of MNT-1 cells were lysed and cDNA was prepared using a Cells to Ct kit (Ambion). Taqman qRT PCR assays (Applied Biosystems) for tyrosinase and MITF was utilized to identify siRNAs that impacted tyrosinase and MITF expression. C) The indicated siRNAs, targeting novel pigmentation genes identified in the MNT-1 screen, were tested for consequences on tyrosinase accumulation in darkly pigmented and moderately pigmented primary human melanocyte cultures 6 days post-transfection. The results presented here is a venn diagram of the data presented in Figure S5 demonstrating that we have identified pigment regulators that differentially impact pigment production in different genetic backgrounds. D) Pharmacological inhibition of Aldh activity impacts tyrosinase protein accumulation. MNT-1 cells (left panel) and primary melanocyte cultures (right panels) were exposed to 5 µM Aldh inhibitors (cyanamide or Angeli's salt) or the tyrosinase inhibitor hydroquinone [15] for 72 hours as indicated. 24 hours post-treatment, cultures were exposed to UV-B at the doses indicated. Tyrosinase and ERK1/2 levels were assessed by immunoblot. MNT-1: Angeli's salt (5 µM), cyanamide (5 µM), or hydroquinone (5 µM); primary melanocytes: Angeli's salt (50 µM), cyanamide (100 µM), hydroquinone (1 µM). E) Aldh inhibitors impair melanogenesis in primary human melanocytes. Darkly pigmented melanocytes were cultured for seven days in the presence of the indicated dosed of cyanamide (cya), vehicle, or PTU. PTU is the most potent currently known *in vitro* pigment inhibitor in primary melanocytes [43]. Subsequently, cells were lysed in CellTiter-Glo and the luminescence and absorbance values were used to calculate inhibition of pigmentation as in Figure 1A.

Initial examination of existing GO annotation data for our pigment regulators exposed a wide variety of cellular processes represented by the validated and candidate hits (Table 1). Therefore, we employed a focused unbiased approach to identify regulators of tyrosinase, the rate limiting enzyme specifying melanogenesis [15] among novel validated genes supporting MNT-1 pigmentation. Relative accumulation of tyrosinase, the melanogenesis transcription factor MITF, and the melanosomal marker protein Melan-A were examined 96 hours post siRNA transfection. Remarkably, over half of the validated pigment genes appear to be required for tyrosinase protein accumulation (Figure 2A, Figure S3). This defect did not appear to be a gross inhibition of cell fate specification, as Melan-A expression was mostly unaffected. In addition, the sub cellular morphology of PMEL17, a melanosome structural protein [27], was normal at the level of immunofluorescence detection (Figure S4). Of those pigment genes impacting tyrosinase accumulation, approximately half appear to act at the level of tyrosinase mRNA accumulation (Table 2), and most of these also impaired MITF mRNA accumulation. Given that tyrosinase is an MITF target gene, the pigmentation genes in this later class may represent action at the level of MITF mRNA. A caveat to this interpretation is our observation that siRNA-mediated turnover of tyrosinase mRNA can also lead to inhibition of MITF gene expression (Figure 2A) through a relationship that remains to be defined. Preliminary studies indicated that this phenotype was not a consequence of siRNA off-target phenomenon (Figure S3).

**Table 2:** Genome-Wide siRNA Screening Identifies Targets That Differentially Impact Tyrosinase and MITF Expression.

Phenotype	Symbol	MITF	Tyrosinase	Melan A
▾ TYR and MITF	TYR	▾ RNA	▾ RNA	NO CHANGE
mRNA	WIPI1	▾ RNA	▾ RNA	▾ PROTEIN
	ALDH1A1	▾ RNA	▾ RNA	NO CHANGE
	ALDH9A1	▾ RNA	▾ RNA	NO CHANGE
	PLEKHA1	▾ RNA	▾ RNA	▾ PROTEIN
	RAB4A	▾ RNA	▾ RNA	▴ PROTEIN
	SERPINB2	▾ RNA	▾ RNA	▴ PROTEIN
	MSRA	▾ RNA	▾ RNA	NO CHANGE
	NPM3	▾ RNA	▾ RNA	▴ PROTEIN
▾ MITF mRNA	ARHGEF11	▾ RNA	▾ PROTEIN	NO CHANGE
▾ TYR protein	ZDHHC9	▾ RNA	▾ PROTEIN	NO CHANGE
	ITPK1	▾ RNA	▾ PROTEIN	▾ PROTEIN
▾ MITF mRNA	AGTR2	▾ RNA	NO CHANGE	NO CHANGE
▾ TYR protein	PPP1R15A	▾ PROTEIN	▾ PROTEIN	NO CHANGE
	ZFYVE1	NO CHANGE	▾ PROTEIN	NO CHANGE
▾ MITF protein	GNG2	▾ PROTEIN	NO CHANGE	NO CHANGE
no change in	EDNRA	NO CHANGE	NO CHANGE	▴ PROTEIN
MITF or TYR	SMARCC2	NO CHANGE	NO CHANGE	NO CHANGE
	FLJ1123	NO CHANGE	NO CHANGE	NO CHANGE
	UROD	NO CHANGE	NO CHANGE	NO CHANGE
	UEV3	NO CHANGE	NO CHANGE	NO CHANGE
	ARL4A	NO CHANGE	NO CHANGE	NO CHANGE
	P66A	NO CHANGE	NO CHANGE	NO CHANGE
	OR4F15	NO CHANGE	NO CHANGE	NO CHANGE

Western blotting and quantitative RT-PCR was used to identify siRNAs that impact tyrosinase, MITF, and Melan-A protein levels or impact tyrosinase and MITF mRNA levels in MNT-1 cells (Figure 2 A, B). siRNAs that significantly impacted the expression of MITF and tyrosinase mRNA as determined by quantitative RT-PCR (p<.05 by student's t-test) and protein as determined by western blotting, or siRNAs that only impacted protein accumulation as determined by western blotting (densitometry values less than 50%) are shown. Genes are sorted into several phenotypes: genes that regulate tyrosinase and MITF protein and mRNA accumulation, genes that regulate MITF mRNA accumulation but only tyrosinase protein accumulation, genes that regulate MITF mRNA accumulation but not tyrosinase or melan-a protein accumulation, genes that regulate protein but not mRNA accumulation of tyrosinase or MITF, and genes that did not impact protein accumulation of tyrosinase or MITF.

While pigmentation in humans is a complex multigenic trait, the degree of genetic variation that contributes to melanocyte autonomous pigment production is unknown. To examine the phenotypic penetrance of novel pigmentation genes, identified in MNT-1 cells, in diverse genetic backgrounds, we employed primary human melanocyte cultures isolated from two different individuals. Remarkably, the majority of targets that regulated tyrosinase expression in MNT-1 cells also impacted tyrosinase expression when depleted from darkly pigmented primary melanocytes (Figure 2C, Figure S5A). Approximately half of these targets also inhibited tyrosinase expression when depleted from moderately pigmented melanocytes (Figure 2C, Figure S5B). These results indicate that the primary screen identified a number of genes that impact pigment production in several different genetic backgrounds. Selective activity of some of these targets in different genetic backgrounds suggests that some of these novel regulators of melanogenesis may play a role in human phenotypic variation. Future large scale studies are required to determine if these genes are differentially expressed in different pigment backgrounds.

For further analyses, we focused on those novel pigmentation genes that impacted tyrosinase expression in all three genetic backgrounds. Among these were two isoforms of aldehyde dehydrogenase, ALDH1A1 and ALDH9A1, well characterized enzymes that regulate ethanol detoxification [28]. A number of chemical inhibitors of these enzymes have been identified [29], and several of these agents are clinically utilized to induce alcohol intolerance during detoxification interventions; presenting an opportunity for pharmacological validation of the contribution of Aldh activity to melanocyte pigmentation. Disulfiram is an Aldh inhibitor that is toxic to melanoma cells via a mechanism that is independent of Aldh inhibition [30]. However, two non-toxic Aldh inhibitors, cyanamide and Angeli's salt [29], inhibited pigmentation and tyrosinase protein accumulation in MNT-1 cells at doses that are equivalent to those required for inhibition of Aldh activity in culture (Figure 2D). For quantifying the impact of compound treatment on pigment accumulation, we used our spectrophotometric-based melanin quantitation assay that couples a CellTiter-Glo assay with a melanin quantitation assay to effectively eliminate compounds that impact cell survival or proliferation. Cyanamide did not appear to impact the viability of MNT-1 cells or primary melanocytes in culture. In addition, these compounds impaired UV-induced tyrosinase expression when tested in primary melanocytes (Figure 2D,E).

**Figure 3:**
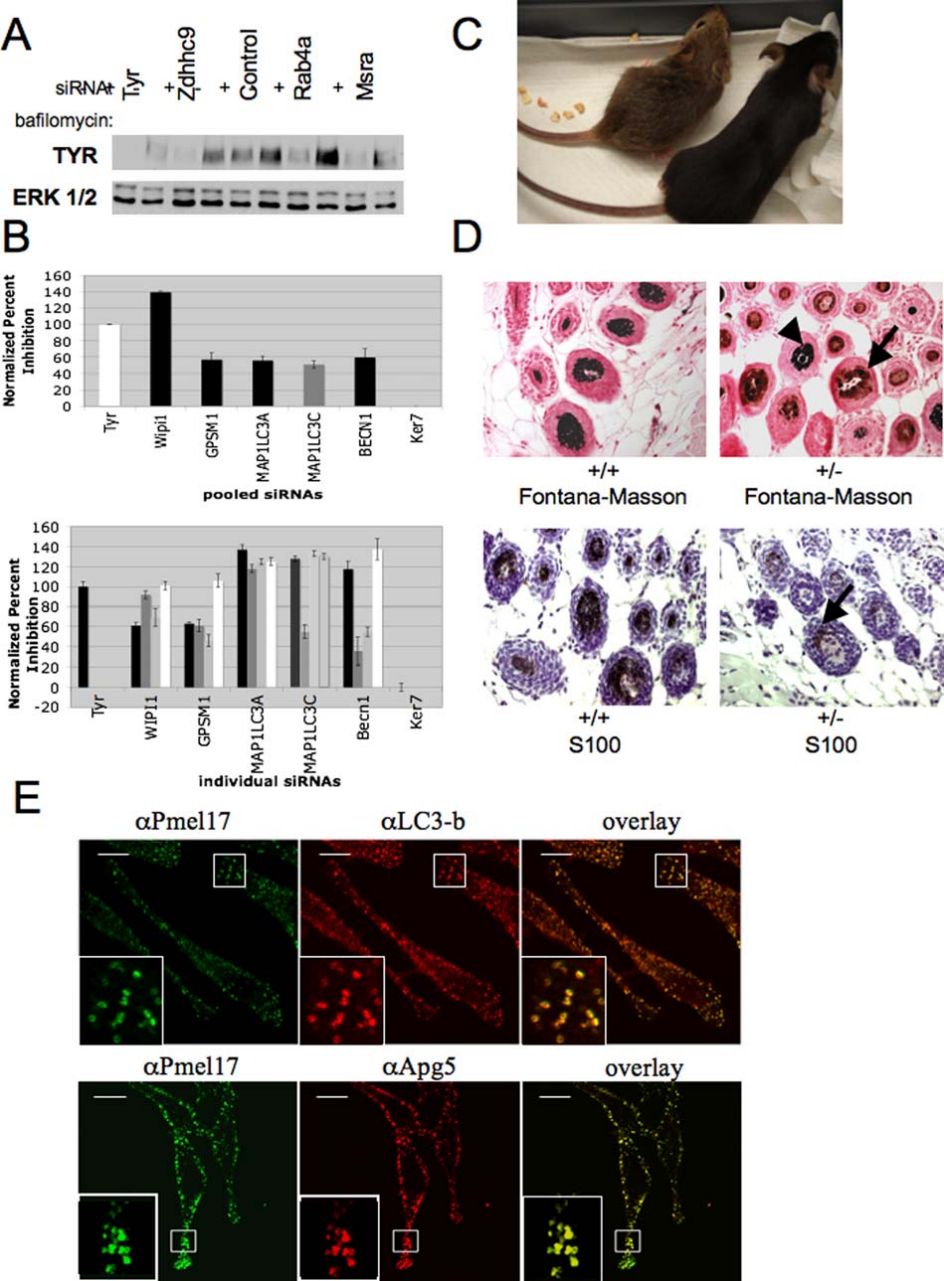
Autophagy is a novel biological process regulating melanin production. A) MNT-1 cells transfected with the indicated siRNAs (50 nM) were incubated in the presence and absence of bafilomycin A1 for 24 hours prior to lyses and analyses of tyrosinase protein accumulation. All results shown are representative of a minimum of three independent experiments. B) MNT-1 cells were transfected with the indicated siRNA pools (50 nM final concentration) or individual siRNAs (75 nM final concentration) targeting putative genes that regulate autophagy identified in the primary screen as described in Figure 1. WIPI1, LC3-C, and GPSM1 were genes identified in the original dataset, while BECN1 and MAP1LC3C are known regulators of autophagy not identified in our initial screening approach. Bars represent mean and s.e.m. for n = 3. Associated p-values (student's t-test) are reported in Table S4. C) Coat color defects in autophagy impaired mice. The coat pigmentation of C57B6 wild type (+/+) (mouse on the right) and heterozygous Beclin 1 mutant littermates (+/−) (mouse on the left) is shown. D) Reduced melanin accumulation in the hair follicles of Beclin1 haploinsufficient mice.. Skin samples from Beclin 1 haploinsufficient mice and wild type littermates were fixed and horizontally sectioned. Upper panels: Fontana-Masson silver staining was used to assess melanin content in the hair follicle (arrow). Detection of staining in wild-type follicles is obscured by accumulation of opaque pigment granules (left panel, and occasional normal follicles in the Beclin+/− background (arrow head)). Sections were counter-stained with aqueous neutral red. Lower panels: the melanoblast marker S100b was used to identify melanocytes in the hair follicle bulb (arrow). Again, staining is obscured in normal follicles due to accumulation of opaque pigment granules. E) MNT-1 cells were fixed and stained with the primary antibodies indicated. Two-photon confocal microscopy was utilized to visualize the colocalization of autophagy and melanosome markers. Representative 0.2 µM confocal slices are shown.

Melanosomes are distinct lysosome-related organelles dependent upon appropriate post-golgi sorting events for delivery of functionalizing ‘cargo’ including tyrosinase [31]. Therefore, impaired accumulation of tyrosinase can be a consequence of misrouting to lysosomes and subsequent hydrolysis in that organelle. To define target genes that may participate in this sorting event, lysosome acidification was inhibited by bafilomycin A1 exposure subsequent to target gene depletion [32]. As shown in Figure 3A, a 24 hour inhibition of lysosome acidification rescued tyrosinase accumulation upon depletion of the small G-protein RAB4A, and the small G-protein palmitoyltransferase ZDHHC9. By contrast, bafilomycin did not restore tyrosinase accumulation upon depletion of MSRA, a protein that can protect against oxidative damage through reduction of methionine sulfoxide. These studies offered preliminary evidence that our screening approach did identify novel genes that impact melanosome trafficking/sorting of melanosome protein cargo.

Gene annotation data was utilized to identify other genes that may regulate melanosome intracellular trafficking/sorting of melanosome protein cargo. Among the panel of validated pigment regulatory genes with phenotypic penetrance in multiple genetic backgrounds was WIPI1 (Figure 2). Wipi1 has been implicated as a human homolog of the yeast autophagy protein ATG18, and is localized to starvation-induced autophagosomes in human cell culture [33]. Two additional autophagy-related proteins, LC3-C and GPSM1/AGS3 were isolated in the primary screen (Table 1). Autophagy, or cellular self-degradation, is a highly conserved cellular pathway that has been associated with cancer formation, neurodegeneration, and aging [34]–[36]. This pathway functions to transport vesicle cargo (autophagosomes) to the lysosome for degradation [35]. Scant evidence currently exists linking autophagy to melanogenesis. Previous studies have documented an abundance of autophagosomes in cells obtained from patients with a disorder of pigmentation (HPS-1) but have hypothesized that their presence is a consequence of the degradation of immature melanosomes within these cells [37]. Other studies have determined that autophagosome components are present in the stage II melanosome, suggesting that parts of the melanosome originate from the autophagosome [38].

Our genome wide siRNA screen directly identified autophagy components as novel regulators of melanogenesis. Validation of these targets by siRNA pool deconvolution supported a functional relationship between autophagosome and melanosome biogenesis (Figure 3B). Furthermore, we found that depletion of two additional components required to trigger autophagosome formation, BECN1 or LC3-A, severely impaired pigment accumulation (Figure 3B). Failure to recover these genes in the primary screen is indicative of the false negative rate inevitably associated with high throughput investigations and illustrates the point that our approach is unlikely to identify all known regulators of melanogenesis. Nonetheless, the validation that both Beclin1 and Lc3 impact pigment accumulation is supporting evidence that autophagy impacts melanogenesis. Consistent with this relationship, heterozygous deletion of the autophagy protein Beclin 1 [39] results in a dramatic coat color defect in mice (Figure 3C, Figure S6). Homozygous null mutations are embryonic lethal, however haploinsufficient animals show an interesting chimeric phenotype with normal and hypopigmented hair follicles. The hypopigmented follicles in these mice contain less pigment in the hair follicle bulb as observed on horizontal sections of the hair follicle. Previous studies have determined that only melanoblasts within the hair follicle unit express S100b protein [40]. To determine if the phenotype observed in beclin1 haploinsuffiicient mice is secondary to an impact of beclin1 depletion on melanoblast survival, we attempted to identify S100+ melanoblasts within the hair follicle in horizontally sectioned skin specimens of wild type and Beclin1 haploinsufficient mice. Consistent with published studies, it was difficult to identify S100 positive cells within the hair follicle of wild type mice secondary to interfering melanin [40]. However, in Beclin1 haploinsufficient mice, we determined that S100+ cells were present in the hair follicle (Figure 3D). Data from our siRNA screen indicated that beclin1 depletion does not impact melanocyte survival. Taken together, our siRNA data and histologic analysis suggests that the phenotype observed in the Beclin1 haploinsuficient mice is not a consequence of impacts of Beclin1 on melanocyte survival but is more likely secondary to the impact of beclin1 on melanosome number or melanin content within the hair follicle. As melanosomes are thought to be lysosome related organelles, autophagic machinery may be required for the functional sorting of melanin synthetic machinery. At the cell autonomous level, we found co-localization of the autophagy proteins LC3 and APG5 and the melanosome markers PMEL17 in mature melanosomes (Figure 3E). Thus molecular components required for autophagosome formation are directly implicated in the biogenesis of melanin, either at the level of melanosome formation or melanosome maturation. When coupled with previous data demonstrating that autophagosomes accumulate in cells defective in melanosome maturation, these results indicate that the autophagy pathway is intimately involved in the process of melanosome maturation [37].

We have utilized an unbiased, high-throughput functional genomics screening platform to identify critical single gene loci that regulate the notoriously complex, highly regulated process of melanogenesis in human cells. Using this approach, we have identified 92 novel genes that impact pigment production in human cells. The convergence of several of these loci directly on the critical rate-limiting enzyme in melanogenesis, tyrosinase, underscores the power of this approach to identify unrecognized genes that are components of even well characterized enzymatic pathways. The complexity of the network controlling tyrosinase expression uniquely parallels the variation in skin color seen in human skin, underscored by the fact that these mechanisms are differentially active in moderately and darkly pigmented melanocytes. The direct identification of novel pigment modulatory agents highlights the utility of genome wide siRNA screening as a translational approach for deriving novel molecular based treatment strategies.

## Methods

### Cell Culture and Reagents

MNT-1 cells were a gift of M. Marks (University of Pennylvania). These cells were cultured in DMEM (Invitrogen) with 15% fetal bovine serum (Hyclone), 10% AIM-V medium (Invitrogen), 1xMEM (Invitrogen) and 1× antibiotic/antimycotic (Invitrogen). Darkly pigmented and moderately pigmented melanocytes were purchased from Cascade Biologics. These cells were cultured in Medium 254 with the melanocyte specific HMGS supplement (Cascade Biologics). Beclin 1 heterozygous mice were obtained from Beth Levine. Angeli's salt was a gift from Pat Farmer. Cyanamide was purchased from Sigma. Bafilomycin A1 was purchased from Tocris Biosciences. The genome wide siRNA library used in these studies was previously described [24]. RPMI 1640 (Invitrogen) was media used for creating lipid oligonucleotide mixtures. All transfections utilized Dharmafect-2 transfection reagent (Dharmacon). For western blotting we utilized the following antibodies: tyrosinase (Santa Cruz Biotechnology, cat # sc-7833), MITF (Santa Cruz Biotechnology, sc-56725), Erk1 (Santa Cruz Biotechnology, sc-94), and Melan-A (Santa Cruz Biotechnology, sc28871). Primary antibody dilutions used in these studies ranged form 1∶200 to 1∶1000 and anti mouse or anti rabbit HRP antibodies (Santa Cruz Biotechnology) were used in the immunoblot analysis. Antibodies used for immunofluorescence are described below. S100B antibody was purchased from DakoCytomation.

### High Throughput Transfection Protocol

High throughput transfection was performed essentially as described [24] with slight modifications. 0.28 pmoles of each siRNA pool in a volume of 30 ul of RPMI was delivered to each of 6 assay plates/master plate using a Biomek FX robotic liquid handler (Beckman Coulter). 0.1 ul of Dharmafect 2 (Dharmacon) in 9.9 ul of RPMI was then delivered to each well using a TiterTek Multidrop. Following a 20–30 minute incubation, 1×10^4^ MNT-1 cells from a trypsin-mediated single-cell suspension were delivered to the siRNA/liposome complexes in a total volume of 200 ul. Plates were incubated for 120 hours at 37°C/5% CO2 after which a Hydra 96 (Robbins-Scientific) was used to removed 100 ul of the medium. 15 ul of CellTiter-Glo Reagent (CTG) (Promega) was delivered to each well and incubated according to manufacturer protocol. Luminescence and absorbance values for each well was recorded using an Envision Plate Reader (Perkin Elmer). Each transfection was performed in duplicate.

### Data Normalization

Raw luminescence values collected from the high throughput screen were normalized to internal reference control samples (cells with no siRNA in wells A1–A8) on each plate to allow for plate-to-plate comparisons. These values were used to normalize absorbance values for each well in the plate, effectively controlling for the impact of each siRNA on cell viability. To normalize for positional variation in the plates secondary to prolonged culture times in the humidified incubator, each well in the plate was normalized to the mean value from all wells in the same location. Mean and standard deviation for each data point and the mean and standard deviation of the entire distribution was calculated. siRNAs that produced absorbance/CellTiter-Glo ratios two standard deviations below the mean were subjected to further analysis.

### Quantitative RT-PCR

4×10^3^ MNT-1 cells were transfected in 96 well plates with 50 nM candidate siRNA using 0.2 ul dharmafect 2 reagent. 48 hours after transfection, cDNA was prepared from transfected cells utilizing a Cells to Ct kit (Ambion) per the manufacterer's protocol. Primers targeting each candidate gene, tyrosinase, actin and MITF were purchased from Applied Biosystems. An aliquot of each cDNA reaction was then added to each Taqman master mix reaction along with the appropriate primer per the manufacturer's protocol (Applied Biosystems). A 7900HT Fast Real-Time PCR System (Applied Biosystems) was utilized to determine Ct values. Values were normalized using actin and analyzed using the relative quantification mathematical model (Pfaffl).

### Drug Treatment

1×10^4^ MNT-1 cells were plated in a 96 well microtiter plate. 24 hours after plating, cells were incubated with vehicle, hydroquinone Angeli's salt, or cyanamide. 48 hours after drug treatment, cell lysates were prepared and subjected to immunoblotting with a tyrosinase and ERK antibody. Similar protocols were utilized in primary melanocytes. Melanocytes were plated in 96 well microtiter plates in the presence of drug or vehicle. 24 hours after drug treatment, melanocytes were treated with UV. Cell lysates were prepared 24 hours after UV treatment and subjected to immunoblotting. In order to measure an impact of cyanamide on pigment production in melanocytes, primary melanocytes were incubated in the presence of vehicle, phenylthiourea, or increasing concentrations of cyanamide. Cells were incubated for an additional 7 days, with one media change on day 4, prior to collection of absorbance and viability values. For bafilomycin experiments, MNT-1 cells were transfected with 75 nM siRNA in 12 well plates. 80 hours after transfection, 25nM bafilomycin was added. 96 hours after transfection cell lysates were prepared and subjected to immunoblotting.

### Immunofluorescence

For immunoflourescence detection of melanosome and autophagy markers, cells were fixed in 2% paraformaldehyde for 1 hour. Coverslips were washed in PBS, cells were permeabilized with 0.1% Triton-X-100 (MNT-1 cells) or 0.4% saponin (primary melanocytes), and blocked in 1% BSA with 0.1% Tween 20. Cells were incubated with the following primary antibodies: Pmel17 (HMB50, Lab Vision Corporation, 1∶100 dilution), Apg5 (Santa Cruz Biotechnology, sc-33210, 1∶50 dilution), and LC3b (Santa Cruz Biotechnology, sc-28266, 1∶50 dilution), for 1 hour. The secondary antibodies used in this study were Alexa Fluor 514 and 594 purchased from Invitrogen. Coverslips were incubated in a 1∶1000 dilution of the corresponding secondary antibody for 1 hour. Confocal images were acquired using a LSM-510 meta confocal multiphoton microscope.

### Mice

Beclin1 haploinsufficient mice were generated as described. [39] All studies involving beclin1 +/+ and beclin1 +/− mice utilized animals that had been backcrossed with C57BL/6J for a minimum of six generations. Representative pictures and figures contained in the manuscript were generated from littermates.

### Immunohistochemistry

Mouse skin sections from four wild type and four beclin 1 haploinsufficient mice were fixed in formalin and paraffin embedded. Haematoxylin and eosin staining were performed following standard protocols. Melanin was stained using the Masson-Fontana technique with a neutral red counterstain [41]. S100 staining was performed as described using an eosin counterstain [42].

## Supporting Information

Figure S1 Genome-wide RNAi screening for novel molecular components of melanogenesis. A) A MNT-1 model for loss-of-function detection of pigmentation genes. MNT-1 cells were transfected with siRNAs targeting tyrosinase using a microtiter-plate based high throughput reverse transfection protocol [24] optimized for this cell line. Inhibition of pigmentation and tyrosinase expression relative to control non-targeting siRNAs is shown. B) MNT-1 pigmented melanoma cells were transfected with 84,920 siRNA duplexes targeting 21,230 genes in a one-well, one-gene reverse transfection format as we have previously described [24]. 120 hrs post transfection, Raw A_405nm_ absorbance values were collected for each well and normalized to internal reference samples on each plate, followed by normalization to the experimental mean for each well calculated from the full data set to control for variations in pigment due to plate and position effects. Similarly adjusted luminescence values from a multiplexed viability assay (CellTiter-Glo) were used to control for cell number, generating “normalized absorbance ratios” for each well (absorbance/cell number; Table S1). The log2 transformation of the average normalized absorbance ratios among replicates is shown for each gene from lowest (hypopigmentation) to highest (hyperpigmentation). Values below 2 standard deviations from the mean are shown in red.
(2.28 MB TIF)

Figure S2 Quantitative RT-PCR was employed to measure the impact of select pooled siRNAs on target mRNA levels. Actin primers were employed to control for mRNA concentrations. Results are representative of three experiments performed in triplicate. A student's t-test was utilized to validate that siRNAs significantly inhibited the expression of the corresponding gene. All of the siRNAs tested in this analysis significantly inhibited the expression of the corresponding gene (p value<.05).
(2.28 MB TIF)

Figure S3 The impact of multiple independent siRNAs, targeting the indicated genes, on tyrosinase protein accumulation was assessed by immunoblot. The impact of a given siRNA on gene expression was quantitated by densitometry (numbers below the corresponding blots). Two or more siRNAs impaired tyrosinase protein expression in all cases examined. Similarly, the impact of multiple independent siRNAs targeting tyrosinase on MITF protein accumulation was assessed by immunoblot. All three siRNAs tested had an impact on MITF expression.
(2.28 MB TIF)

Figure S4 MNT-1 cells transfected with the indicated siRNAs were immunostained with anti-Pmel antibodies to detect melanosomes 4 days post transfection. Representative micrographs are shown.
(2.28 MB TIF)

Figure S5 Identified pigment regulators differentially regulate tyrosinase expression in different genetic backgrounds. A) The indicated siRNAs targeting novel pigmentation genes identified in the MNT-1 screen were tested for consequences on tyrosinase protein accumulation in darkly pigmented primary human melanocyte cultures 6 days post transfection. A non-targeting siRNA was used as a transfection control (Control). ERK1/2 is shown as a loading control. B) The indicated siRNAs, targeting novel pigmentation genes identified in the MNT-1 screen, were tested for consequences on tyrosinase protein accumulation in moderately pigmented primary human melanocyte cultures 6 days post transfection as in (A). A venn diagram in Figure 2 identifies sets of genes that differentially impact tyrosinase accumulation in different genetic backgrounds.
(2.28 MB TIF)

Figure S6 Autophagy deficient mice have coat color defects. Photograph depicts the coat color of 4 wild type and 4 beclin 1 haploinsufficient littermates. Note that no pigmentary defects are noted on the nose which argues against defects in melanoblast migration. Mice are of similar size. Additionally, no pigment defects were noted on the belly.
(2.28 MB TIF)

Table S1 Genome-wide siRNA dataset. MNT-1 pigmented melanoma cells were transfected with 84,920 siRNA duplexes targeting 21,230 genes in a one-well, one-gene reverse transfection format as we have previously described [24]. 120 hrs post transfection, Raw A_405nm_ absorbance values were collected for each well and normalized to internal reference samples on each plate, followed by normalization to the experimental mean for each well calculated from the full data set to control for variations in pigment due to plate and position effects. Similarly adjusted luminescence values from a multiplexed viability assay (CellTiter-Glo) were used to control for cell number, generating “normalized absorbance ratios” for each well (absorbance/cell number). Data for each individual absorbance ratio, the mean, and standard deviation is shown for all of the genes examined. Additionally gene ontology information for each gene, accession number, and chromosome position is indicated.
(10.32 MB XLS)

Table S2 Genome wide siRNA screen identifies known regulators of melanogenesis. 127 mouse coat color genes have been identified to date and 68 of these genes have human homologues. To validate that our screening approach could identify known regulators of melanogenesis, we identified genes in the coat color database whose corresponding siRNAs significantly inhibited pigment production in our assay. SiRNAs corresponding to 13 of the 68 genes in this database significantly impacted pigment production. Normalized pigment inhibition scores and standard deviations are listed for each of these genes.
(0.02 MB XLS)

Table S3 siRNA sequences that significantly inhibited pigment production in our siRNA Screen. The corresponding sequences for each of the four siRNA duplexes that collectively inhibited pigment production in our siRNA library is shown.
(0.06 MB XLS)

Table S4 Individual and pooled siRNAs Directed at novel pigment regulators significantly impact pigment production. Individual and pooled siRNAs directed towards 35 of the novel regulators of pigment production identified in our genome wide screen were retested to examine their impact on pigment production (retest data for 32 genes is shown in figure 1, while retest data for 3 remaining genes is shown in Figure 3). A student's t-test was used to determine if these siRNAs were able to significantly inhibit pigment production when compared to a control siRNA (Ker7). 4 of the siRNA pools retested did not significantly inhibit pigment production. Two of the four oligos retested for all of the other genes examined. Underlined numbers correspond to p-values less than .05, while bold numbers correspond to p-values less than .01. The results are representative of a minimum of three experiments done in triplicate.
(0.04 MB XLS)
