# Supplementary figures and images for: Genome-Wide siRNA-Based Functional Genomics of Pigmentation Identifies Novel Genes and Pathways That Impact Melanogenesis in Human Cells
Source: PLoS Genet. 2008 Dec 5;4(12):e1000298. doi: 10.1371/journal.pgen.1000298 (PMC2585813; doi:10.1371/journal.pgen.1000298)

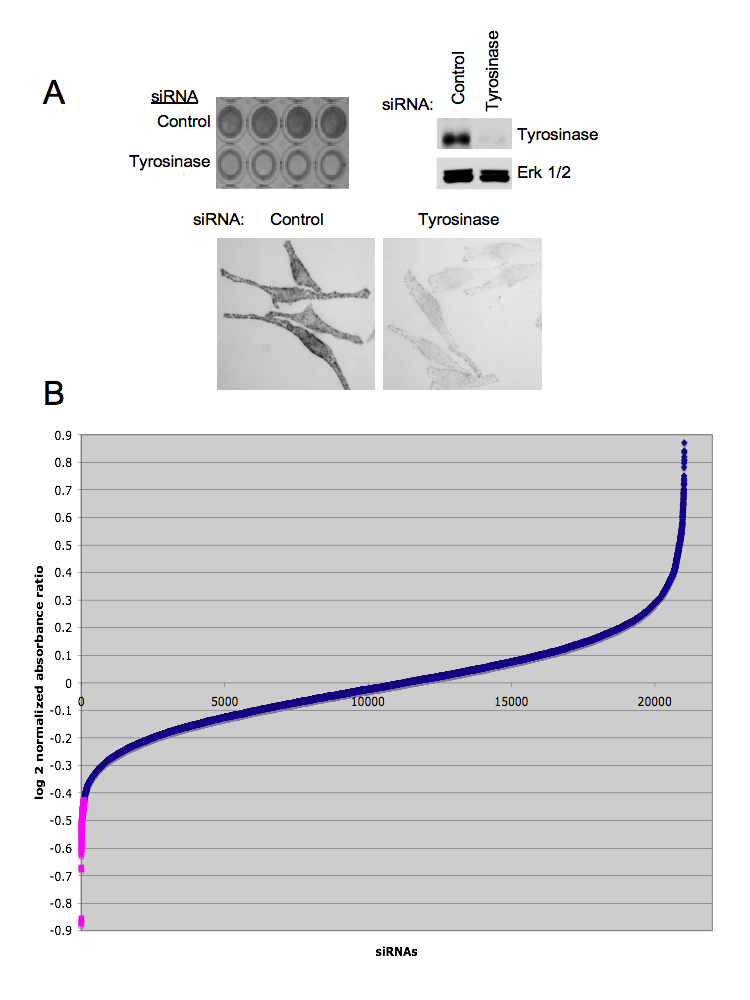

Supplement: Figure S1 — Genome-wide RNAi screening for novel molecular components of melanogenesis. A) A MNT-1 model for loss-of-function detection of pigmentation genes. MNT-1 cells were transfected with siRNAs targeting tyrosinase using a microtiter-plate based high throughput reverse transfection protocol [24] optimized for this cell line. Inhibition of pigmentation and tyrosinase expression relative to control non-targeting siRNAs is shown. B) MNT-1 pigmented melanoma cells were transfected with 84,920 siRNA duplexes targeting 21,230 genes in a one-well, one-gene reverse transfection format as we have previously described [24]. 120 hrs post transfection, Raw A405nm absorbance values were collected for each well and normalized to internal reference samples on each plate, followed by normalization to the experimental mean for each well calculated from the full data set to control for variations in pigment due to plate and position effects. Similarly adjusted luminescence values from a multiplexed viability assay (CellTiter-Glo) were used to control for cell number, generating “normalized absorbance ratios” for each well (absorbance/cell number; Table S1). The log2 transformation of the average normalized absorbance ratios among replicates is shown for each gene from lowest (hypopigmentation) to highest (hyperpigmentation). Values below 2 standard deviations from the mean are shown in red. (2.28 MB TIF) [file pgen.1000298.s001.tif]

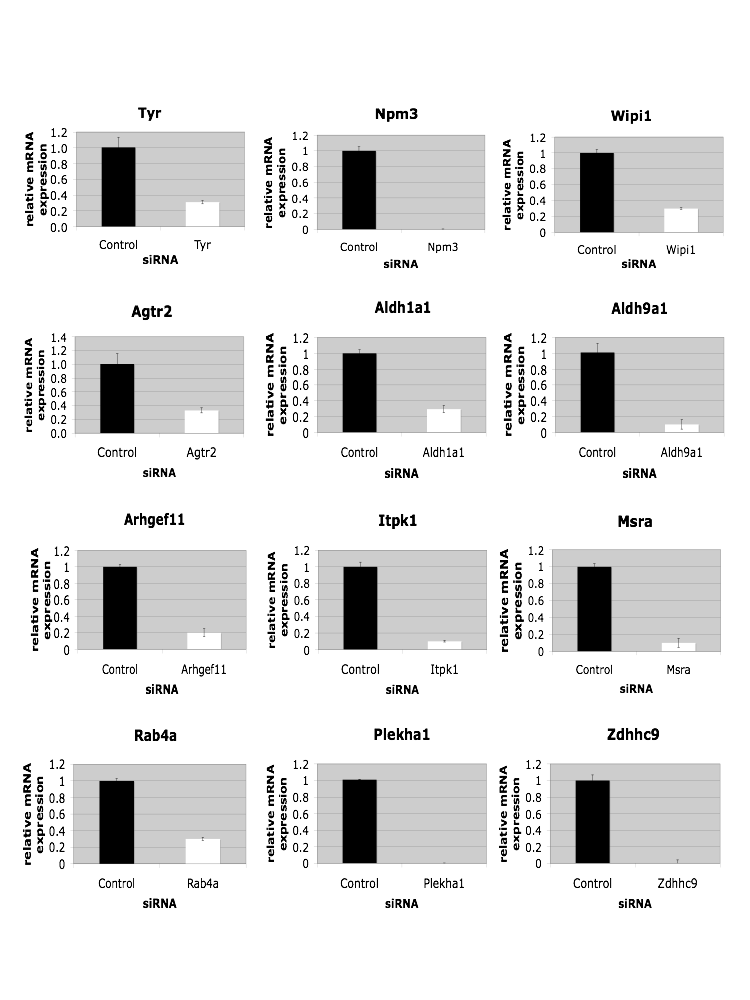

Supplement: Figure S2 — Quantitative RT-PCR was employed to measure the impact of select pooled siRNAs on target mRNA levels. Actin primers were employed to control for mRNA concentrations. Results are representative of three experiments performed in triplicate. A student's t-test was utilized to validate that siRNAs significantly inhibited the expression of the corresponding gene. All of the siRNAs tested in this analysis significantly inhibited the expression of the corresponding gene (p value<.05). (2.28 MB TIF) [file pgen.1000298.s002.tif]

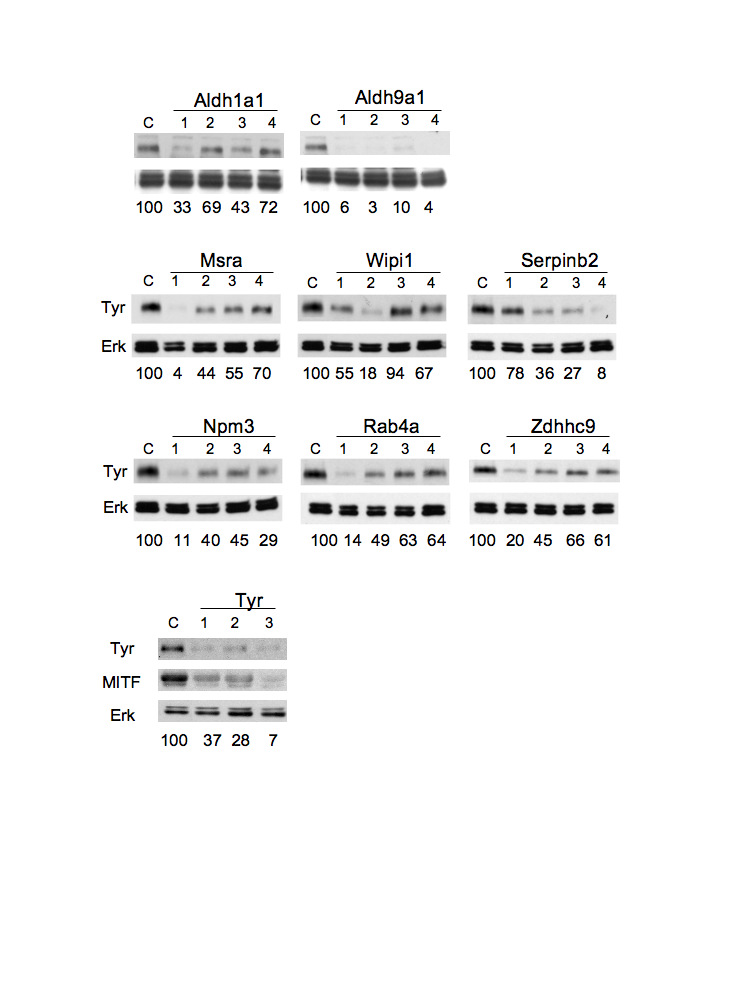

Supplement: Figure S3 — The impact of multiple independent siRNAs, targeting the indicated genes, on tyrosinase protein accumulation was assessed by immunoblot. The impact of a given siRNA on gene expression was quantitated by densitometry (numbers below the corresponding blots). Two or more siRNAs impaired tyrosinase protein expression in all cases examined. Similarly, the impact of multiple independent siRNAs targeting tyrosinase on MITF protein accumulation was assessed by immunoblot. All three siRNAs tested had an impact on MITF expression. (2.28 MB TIF) [file pgen.1000298.s003.tif]

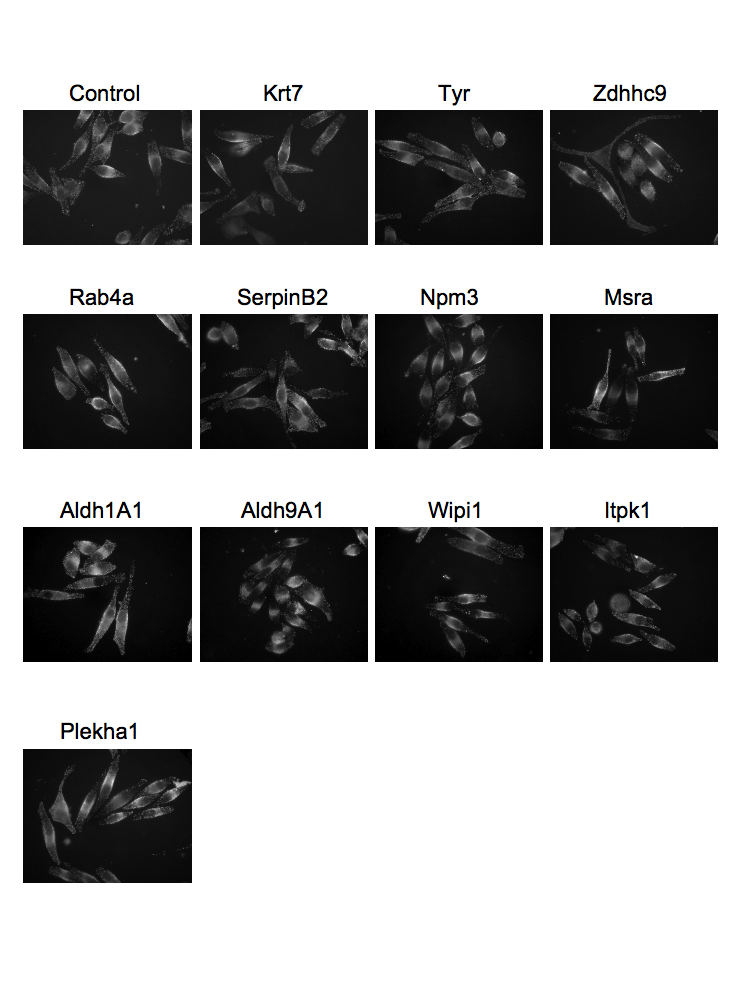

Supplement: Figure S4 — MNT-1 cells transfected with the indicated siRNAs were immunostained with anti-Pmel antibodies to detect melanosomes 4 days post transfection. Representative micrographs are shown. (2.28 MB TIF) [file pgen.1000298.s004.tif]

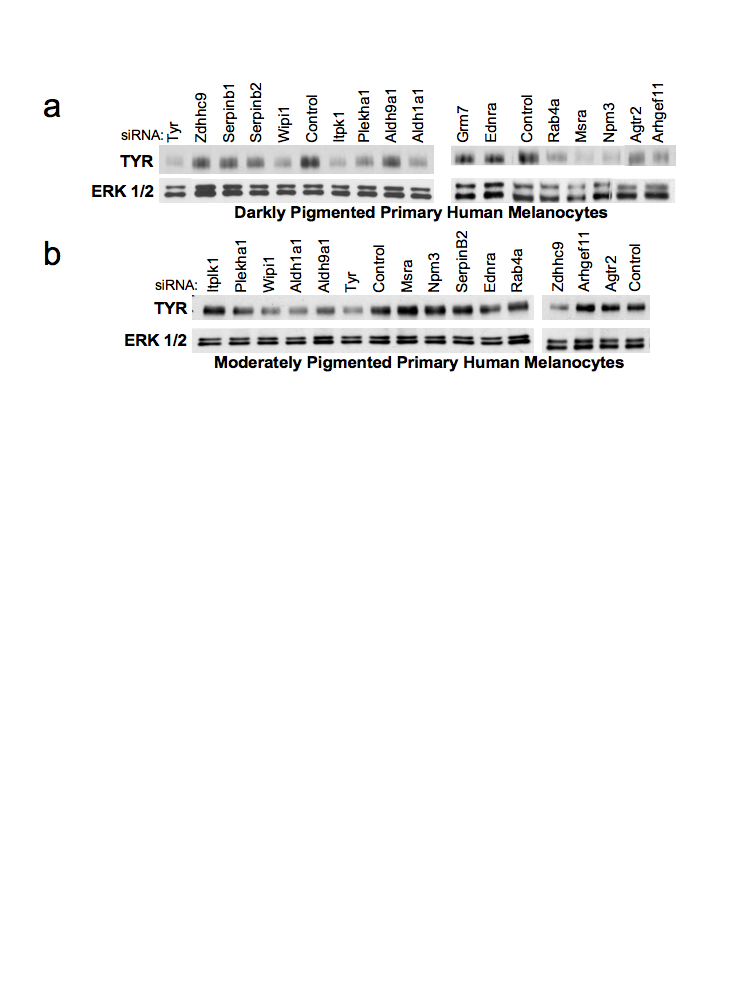

Supplement: Figure S5 — Identified pigment regulators differentially regulate tyrosinase expression in different genetic backgrounds. A) The indicated siRNAs targeting novel pigmentation genes identified in the MNT-1 screen were tested for consequences on tyrosinase protein accumulation in darkly pigmented primary human melanocyte cultures 6 days post transfection. A non-targeting siRNA was used as a transfection control (Control). ERK1/2 is shown as a loading control. B) The indicated siRNAs, targeting novel pigmentation genes identified in the MNT-1 screen, were tested for consequences on tyrosinase protein accumulation in moderately pigmented primary human melanocyte cultures 6 days post transfection as in (A). A venn diagram in Figure 2 identifies sets of genes that differentially impact tyrosinase accumulation in different genetic backgrounds. (2.28 MB TIF) [file pgen.1000298.s005.tif]

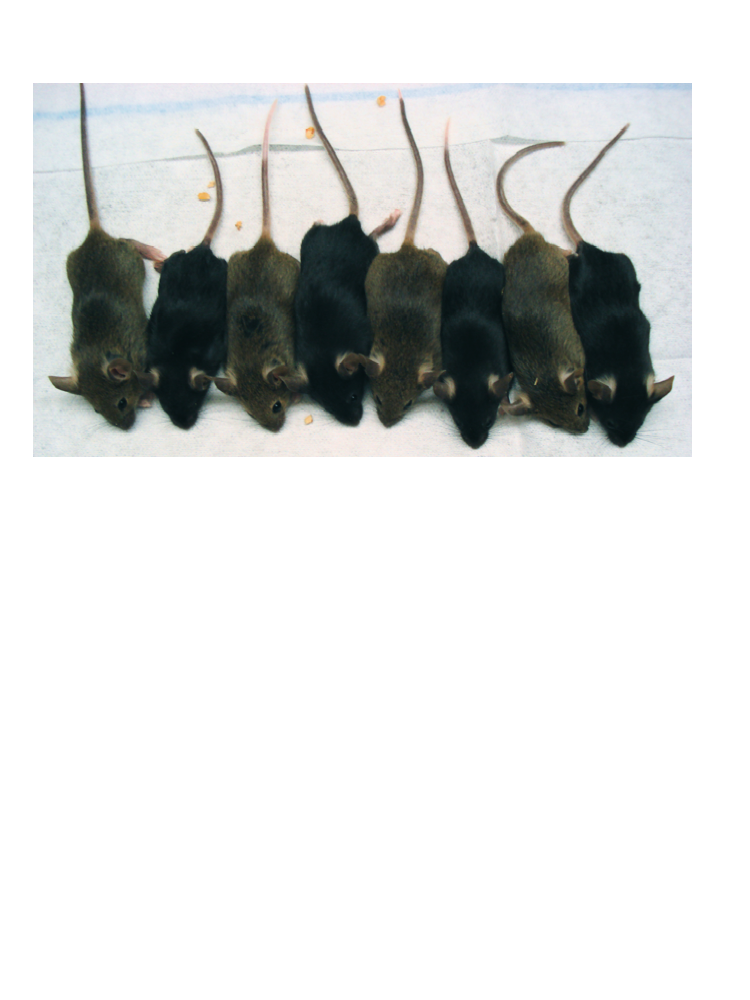

Supplement: Figure S6 — Autophagy deficient mice have coat color defects. Photograph depicts the coat color of 4 wild type and 4 beclin 1 haploinsufficient littermates. Note that no pigmentary defects are noted on the nose which argues against defects in melanoblast migration. Mice are of similar size. Additionally, no pigment defects were noted on the belly. (2.28 MB TIF) [file pgen.1000298.s006.tif]
